# Two Sides of the Coin: The Roles of Adverse Childhood Experiences and Positive Childhood Experiences in College Students’ Mental Health

**DOI:** 10.1177/08862605231220018

**Published:** 2023-12-18

**Authors:** Madhav Bhargav, Lorraine Swords

**Affiliations:** 1 Trinity College Dublin, Ireland

**Keywords:** adverse childhood experiences, college students, mental health, positive childhood experiences, resilience, suicide

## Abstract

Several studies have established a link between adverse childhood experiences (ACEs) and mental health issues in college students. However, less is known about how positive childhood experiences (PCEs) may promote mental health and well-being, and potentially act as a buffer in the relationship between risk exposure and poor outcomes. This study investigates how ACEs and PCEs relate to college students’ mental health (*N* = 321), within the framework of Resiliency Theory with specific attention focus on the compensatory and the protective factors models. Three key hypotheses were examined using quantitative data collected by way of an online anonymous survey: (1) ACEs will predict poorer mental health outcomes; (2) PCEs will predict better mental health outcomes and will lessen the negative effects of ACEs on mental health outcomes (compensatory factor model), and (3) PCEs will moderate the association between ACEs and mental health outcomes so that the relationship will be weaker among participants with higher PCEs (protective factor model). Findings supported each of these hypotheses and are important for our understanding of the long-term mental health correlates of ACEs and PCEs among college students. Our study underscores the importance of promoting PCEs while also underscoring the necessity of proactively preventing ACEs. Practical implications are discussed in relation to improving assessments of student needs and providing targeted interventions for those at risk.

## Introduction

A comprehensive body of research has accumulated over the past 25 years establishing the detrimental effects of experiencing multiple adverse childhood experiences (ACEs) on later mental and physical health (Felitti et al., 1998; Lee et al., 2022). ACEs are defined as a set of highly correlated negative and potentially traumatic experiences involving (1) abuse (emotional, physical, and/or sexual); (2) neglect (emotional and/or physical); and (3) household dysfunction (e.g., witnessing domestic violence between or among caregivers, family members with mental illness, parental divorce/separation, and/or parental incarceration). The cumulative burden of such chronic stressors and damaging life events can have persistent and pervasive consequences for development by way of changes they exert on the nervous, endocrine, and immune systems (Danese & McEwen, 2012). This allostatic load, or overload, has in turn been associated with a range of negative health outcomes (Guidi et al., 2021).

Research has also demonstrated how favorable early life circumstances or experiences shape brain development and influence the functioning of multiple physiological systems to impact favorably upon health and well-being across the life span (Lamb & Lerner, 2015; Masten & Barnes, 2018). However, standardized measures of positive childhood experiences (PCEs) in research exploring the relationship with individuals’ long-term health and well-being have only recently been adopted. PCEs are conceptual “short lists” of positive early influences and include healthy attachment bonds with caregivers, effective parenting behaviors, meaningful beliefs, and other resources within communities and societies, including close and supportive relationships with family and friends (Bethell et al., 2019). As with ACEs, research suggests that cumulative PCEs can also affect mental and physical health in adulthood, although in a positive way. For example, a recent study conducted in China noted an inverse dose-response relationship between cumulative PCEs exposure and risk of depression (Qu et al., 2022). Research has also explored how PCEs can act as protective or promotive factors in mitigating the long-term effects of early adverse experiences (Crandall et al., 2019; Walsh et al., 2020).

### Risk and Resilience

Not all children and adolescents who experience ACEs have poor long-term mental and physical health outcomes (Moore & Ramirez, 2016). Children may successfully cope with exposure to potentially traumatic experiences and continue on a positive life course trajectory (Fergus & Zimmerman, 2005). This process of overcoming adversity is defined as resilience, the ability to bounce back from, and flexibly adapt to, the changing demands or outcomes of stressful experiences (Block & Kremen, 1996; Garmezy et al., 1984; Hu et al., 2015; Zimmerman et al., 2013). Resilience researchers who adopt a bio-ecological or social-ecological approach in their work consider multisystemic person–environment reciprocal processes involving individual, social, and situational promotive factors that have the potential to buffer against risk factors and interrupt a negative life course (Fergus & Zimmerman, 2005; Ungar et al., 2013). Assessing positive experiences in childhood in addition to childhood adversity can inform our understanding of how resilience and risk processes operate and interact across the lifespan, and in different contexts (Merrick & Narayan, 2020).

**Figure 1.**
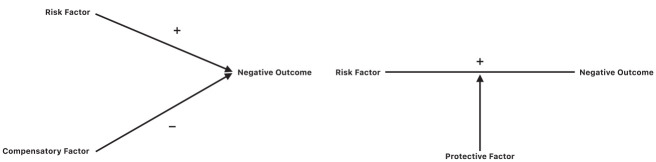
The compensatory and protective factor models of resilience (Fergus & Zimmerman, 2005).

Two of the most commonly studied frameworks that guide our thinking about how promotive factors may counteract or guard against the negative effects of risks are the compensatory and protective models of resilience (Zimmerman, 2013). The compensatory model proposes that promotive factors offset the effects of risk factors and have the opposite effect on an outcome, operating directly and independently to risks. The protective model of resilience suggests that interactions between promotive and risk factors reduce the probability of a negative outcome as promotive factors moderate the negative effects of the exposure to risk (O’Leary, 1998). See Figure 1 depicting these two models of resilience.

### Impact of Childhood Experiences on College Students’ Mental Health

Approximately 75% of lifelong mental health disorders arise by the age of 24 (Kessler et al., 2005). Research that examines the role of ACEs in mental disorders among students is limited but suggests a number of possible mediating variables. For example, in the United States, Karatekin and Ahluwalia (2020) noted that students with a greater number of accumulated ACEs had greater levels of stress and lower levels of social support. ACEs, stress, and lower levels of support all predicted poorer mental health. Recent research from Ireland reports how a greater number of ACEs predicted suicidal ideation among students both directly and indirectly, through their negative effect on perceived burdensomeness, belongingness, and psychological distress (Bhargav & Swords, 2022).

Few studies have explored how PCEs might influence young adults’ mental health or interact with ACEs to reduce risk outcomes, though among college students’ positive early life experiences have been found to significantly predict self-esteem and resilience (Kocatürk & Çiçek, 2021) and act as an independent protective factor against poor mental health after accounting for ACEs (Xu et al., 2022). This gap in the literature is an important to address in order to further clarify the association and interrelated nature of risk and promotive factors in determining young adults’ mental health functioning.

### The Present Study

The primary goal of the present study is to apply the compensatory and protective models of resilience to help explain how ACEs and PCEs relate to mental health and the construction of resilience in a young college-going population. Data pertaining to ACEs in this research was based on the “ACE-pyramid” (Felitti et al., 1998) conceptual model that offers a framework for studying and accounting for the co-occurrence and interrelatedness of childhood adversities and their influence on health and life conditions across the lifespan. Therefore, the ACE questions in this study drew upon the original ACE questionnaire (ACE-Q) (Felitti et al., 1998), which comprised seven categories of stressors presented under three superordinate categories of “abuse,” “neglect,” and “household dysfunction.” Two additional questions reflecting experiences of domestic abuse and illness within the family were added in order to capture a broader conceptualization of ACEs (Finkelhor, 2020).

Within the context of the compensatory model, it is anticipated that, irrespective of an individual’s number of ACEs, their PCEs will have an independent, direct, and positive association with their mental health functioning. Furthermore, PCEs will protect against poor mental health and promote well-being, weakening the effects of ACEs on mental health functioning. The protective factors model notes that promotive resources serve to moderate the relationship between risk factors and outcomes, and so it is anticipated that the relationship between ACEs and mental health functioning will be weaker among those with higher PCEs scores.

Three primary hypotheses are proposed to be tested with a sample of college students. The specific mental health outcomes under investigation across all hypotheses are (1) psychological distress, (2) suicide ideation, and (3) resilience.

1. ACEs will predict poorer mental health outcomes.
2. To test the compensatory model, it is hypothesized that: (1) PCEs will predict better mental health outcomes and (2) PCEs will lessen the negative effects of ACEs on mental health outcomes.
3. To test the protective factor model, it is hypothesized that PCEs will moderate the association between ACEs and mental health outcomes so that the relationship will be weaker among participants with higher PCEs.

## Methods

### Participants

Participants were 321, mostly European Union (85%), college students aged from 18 to 21 years (*n* = 178) and 22 to 25 years (*n* = 145). Approximately two-thirds of participants identified as heterosexual (65.4%), whereas the remaining third (34.6%) identified as non-heterosexual (e.g., gay/lesbian, bisexual, or asexual) or declined to report this information. The majority of participants identified themselves as female (78.8%). Just over 18% identified as male, and 3.1% identified as genderqueer, trans male, trans female, or declined to report this information. See Table 1 for more information on demographics. Online consent was obtained from all the participants.

**Table 1.** Demographic Characteristics of College Student Sample.

Characteristic	*n*	%
Age		
18–21 years	176	54.8
22–25 years	145	45.2
Sexual orientation		
Heterosexual/Straight	210	65.4
Bisexual	66	20.6
Gay/Lesbian	32	10
Prefer not to answer	9	2.8
Asexual	2	0.6
Do not specify as any	2	0.6
Gender		
Female	253	78.8
Male	58	18.1
Genderqueer	5	1.6
Trans female	3	0.9
Trans male	1	0.3
Prefer not to answer	1	0.3
Nationality status		
EU	272	84.7
Non-EU	49	15.3

*Note*. EU = European Union.

### Ethics

This study was approved by the School of Psychology Research Ethics Committee—approval: SPREC092020-05. Data were collected through an anonymous online cross-sectional quantitative questionnaire, and all participants took part voluntarily. Informed consent was obtained electronically after the participants had received a detailed introduction to the study.

### Measures

#### Adverse Childhood Experiences

Cumulative exposure to ACEs was assessed using an adapted version of the Early Adverse Experiences Questionnaire (Felitti et al., 1998), which assess the presence or absence of maltreatment (e.g., emotional abuse, physical abuse, sexual abuse) and household dysfunction (e.g., substance abuse or domestic violence) before age 18 years. Questions are phrased such as “Did a parent or other adult in the household often or very often . . . Swear at you, insult you, put you down, or humiliate you?” (emotional abuse item), or “. . . act in a way that made you afraid that you might be physically hurt?” (physical abuse item). Two additional questions were added to the original questionnaire in order to capture a broader conceptualization of ACEs (Finkelhor, 2020). These items were “Were your parents or stepparents arguing, yelling, and angry at one another a lot of the time?” and “Did your parents, brother or sister, or best friend suffer a ‘very bad illness’ or ‘very bad accident’ where they had to be in the hospital for a long time?.” Participants responded with either *Yes* or *No* answers where *Yes* = 1 and *No* = 0. The number of experiences reported by each participant was summed for a total ACEs score ranging from 0 to 12. ACEs questions have been used in several studies with young adults globally (e.g., Karatekin, 2018), reporting acceptable reliability and validity.

#### Positive Childhood Experiences

All participants completed the 10-item PCE checklist of positive experiences between the age of 0 and 18 years (Narayan et al., 2018). Items are related to perceived relational and internal safety and security (e.g., at least one safe caregiver, beliefs that gave comfort), and positive and predictable quality of life (e.g., enjoyment of school, regular meals, and bedtime). Questions such as “Did you have at least one caregiver with whom you felt safe?,” “Did you have at least one teacher who cared about you?,” and “Did you have opportunities to have a good time?” were asked. Participants responded with either Yes or No answers where Yes = 1 and No = 0. The number of experiences reported by each participant was summed for a total PCEs score ranging from 0 to 10. The PCEs scale has demonstrated high test–retest reliability (r = 0.80, p < .01), good predictive validity for later mental health problems, and good cultural generalizability (Narayan et al., 2018).

#### Psychological Distress

The 10-item Clinical Outcomes in Routine Evaluation (Barkham et al., 2015) tool assesses anxiety, depression, trauma, physical problems, general functioning, and risk to self over the past week. Participants rate each item on a 5-point scale ranging from 0 (*Not at all*) to 4 (*Most or All the time*). For the final score, all items are added together to get the Clinical Score, with higher scores indicating higher distress, anxiety, and depression. Questions include “I have felt I have someone to turn to for support when needed,” and “Talking to people has felt too much for me.” The CORE 10 internal reliability (alpha) is .90 and the score for the CORE-10 correlated with the CORE-OM (the longer scale that the CORE-10 is derived from) is .94 in a clinical sample and .92 in a non-clinical sample. Barkham suggested that score range between 11 and 14 should be considered as mild psychological distress, score range between 15 and 19 as moderate psychological distress and scores above 20 as severe psychological distress. The Cronbach’s α for the current sample was .85.

#### Suicidal Ideation Scale

This 10-item Suicidal Ideation Scale (Rudd, 1989) measures Suicide Ideation (SI) and behaviors. The Suicidal Ideation Scale (SIS) includes a 6-item subscale measuring resolved suicide preparations and behaviors such as “I have made attempts to kill myself” and a 4-item subscale measuring suicidal desire for example “I feel life just isn’t worth living.” Participants rate each item on a 5-point scale ranging from 1 (*never*) to 5 (*always*). Items are summed, with higher scores indicating a more severe risk for suicide. Total scores range from 10 to 50. Strong internal consistency (Cronbach’s α = .86) and concurrent validity have been reported (Rudd, 1989). Cronbach’s α for the current sample was .89.

#### Resilience

Participants’ resilience was measured using the Brief Resilience Scale (BRS) developed by Smith and colleagues (Smith et al., 2008). The BRS consisted of 6 items identifying one’s ability to bounce back from stress. Items 1, 3, and 5 were positively worded, and items 2, 4, and 6 were negatively worded. The BRS is scored by reverse coding items 2, 4, and 6 and finding the mean of the 6 items. Participants were asked to indicate the extent to which they agree with statements such as “I tend to bounce back quickly after hard times” on a 5-point Likert scale (1 *=* *strongly disagree* to 5 *=* *strongly agree*). Internal consistency of BRS is good, ranging from .80 to .91 (Smith et al., 2008). Smith et al. noted that the score range between 1 and 2.99 are considered as low resilience, 3 and 4.30 as normal resilience, and lastly, 4.31 and 5 as high resilience. In the present study, internal reliability was .83.

### Procedure

The data was collected between February and March 2021. Participants were recruited via (1) Students Union weekly emails and (2) advertisements for this study posted on social media (Twitter and Facebook). Given the social media based recruitment strategy, the data were screened for “bots” by tracking study time stamps for unusual or illogical dates and times (e.g., groups of participants beginning and ending the survey at the exact same time) and checking for respondents who completed the survey at an impossibly fast speed. Participants who gave web-based consent anonymously completed all questionnaires using a secure survey website called Qualtrics. Participants took between 8 and 12 minutes to complete the survey. At the end, participants were thanked for their time and directed to the debrief sheet that included a list of mental health services and the researcher’s contact details, should they have any follow-up questions about the research.

### Data Analysis Plan and Preparation

Data were analyzed using SPSS 27 (SPSS Inc., Chicago, IL, USA) and R (R Core Team, 2022; version 4.2.2). As gender differences and sexual orientation are frequently reported about mental health functioning in adolescents and young adults, participants’ gender and their sexual orientation were controlled in all analyses by dummy-coding the gender and sexual orientation variables.For gender, we dummy-coded the variable into females and others, with female as the reference category (coded as *cisgender women* = 1 and *others including cisgender men, transgender, and gender diverse individuals* = 0). For sexual orientation, we dummy-coded the variable into heterosexual and others, with heterosexual as the reference category (*coded as heterosexual* = 1 and *others including gay, lesbian, bisexual, asexual* = 0).

For hypothesis one, three separate unadjusted regression models were designed to independently assess the effect of ACEs on outcome variables (H1). Analyses for hypotheses two and three were guided by Crandall et al. (2019). Hypotheses two aimed to test the compensatory model of resilience and first involved conducting three separate unadjusted regression models so as to independently assess the effects of PCEs on the outcome variables; second, PCEs were added as an independent variable to the regression models assessing how ACEs predict mental health outcomes (H2). To test the protective factor model of resilience for hypothesis three, we stratified the sample by individual’s PCE score (*mean* = 7.59) into two groups using a mean split (≤8.0 vs. >8.0) and conducted regression analyses for each group to examine the impact of ACEs on the mental health outcomes (H3).

## Results

### Preliminary Analysis: PCEs, ACEs, and Mental Health Functioning

PCEs were common among college students with a mean of 7.59 out of a possible 10. Approximately 27% of the participants (*n* = 88) reported a top score of 10, and 90% responded that they had experienced at least four or more PCEs (*n* = 294). Childhood adversities were also common, with students endorsing a mean of 2.94 ACEs out of a possible 12. Although a quarter (25.2%) of students reported experiencing no adversities from the list during their childhood, 35.2% endorsed between 1 and 3 ACEs, and 39.6% endorsed between 4 and 12 ACEs. Forty-three percent of the sample population reported experiencing some type of abuse (physical, sexual, or emotional), and approximately 38% of the sample population reported some type of neglect (emotional or physical). Moreover, approximately 65% of respondents reported some type of a household dysfunction such as parental divorce.

The mean score of 18.25 on the CORE-10 indicates that on average students displayed moderate levels of psychological distress (Marriott et al., 2019). Regarding SI, the current sample established a mean of 16.38. Based on results from the initial SIS validation study (Rudd, 1989), scores greater than one standard deviation above the mean (SIS total score of 15 or greater) are proposed to be indicative of serious SI (Luxton et al., 2011). Lastly, the mean score of 2.69 for the Brief Resiliency Scale suggests a moderate level of resiliency within the sample (Smith et al., 2008). See Table 2 for more descriptive information on the data.

**Table 2.** Descriptive Statistics—Achieved mean, Standard deviation, and Min–Max of the achieved scores.

Variables	*N*	Mean	*SD*	Min	Max
PCEs (range 0–10)	321	7.59	2.33	0	10
ACEs (range 0–12)	321	2.94	2.62	0	9
Resiliency	321	2.69	0.77	1	4
Psychological distress	321	18.25	7.87	1	39
Suicide ideation	321	16.38	9.14	10	50

*Note*. ACE = adverse childhood experience; PCE = positive childhood experience; *SD* = standard deviation.

### Preliminary Analysis: Pre-Hypotheses Testing

**Figure 2.**
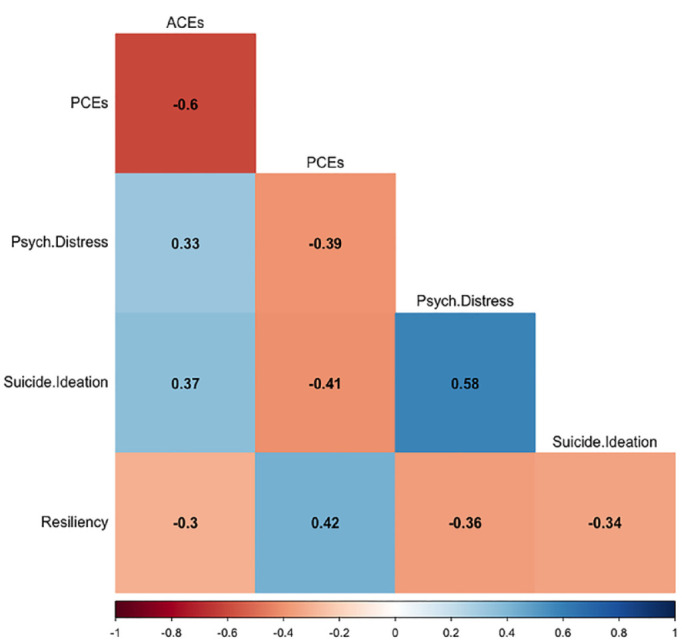
Pearson’s correlation matrix for all key study variables.

Pearson correlation coefficient tests was computed to assess the linear relationships between the key variables (see Figure 2). There were significant positive correlations between ACEs and psychological distress (*r* = 0.329; *p* = .001), and suicide ideation (*r* = 0.368; *p* = .001). ACEs were also found to be significantly negatively associated with resiliency (*r* = −0.301; *p* = .001). In contrast, a significant positive correlation was found between PCEs and resiliency (*r* = 0.423; *p* = .001), whereas significant negative relationships were noted between PCEs and psychological distress (*r* = −0.391; *p* = 0.001), and suicide ideation (*r* = −0.408; *p* = .001).

Hypothesis one: ACEs will predict poorer mental health outcomes

To test hypothesis one, three independent regression models were conducted with the estimates provided in Table 3. The model suggested that ACEs scores predicted greater psychological distress (*p* < .001), suicide ideation (*p* < .001), and lower levels of resilience (*p* < .001).

**Table 3.** Compensatory Model: Regression Results for Mental Health Outcomes in the Sample of College Students (*N* = 321).

Outcome variable	PCEs	ACEs	
		Unadjusted	Adjusted^a^
Psychological distress	−0.39^***^	0.32^***^	0.15^*^
Suicide ideation	−0.37^***^	0.34^***^	0.19^**^
Resiliency	0.41^***^	−0.29^***^	−0.07

*Note*. Gender and sexual orientation controlled in all analyses: Coded as *females* (1) and *other* (0); *heterosexual* (1) and *other* (0). ACE = adverse childhood experience; PCE = positive childhood experience.

a In adjusted models, PCEs were added as an independent variable.

* *p* < .05. **p < .01. ***p < .001.

Hypothesis two (a): PCEs will predict better mental health outcomes

To test the first part of hypothesis two, three independent regression models were conducted with the estimates provided in Table 3. PCEs scores predicted greater resilience (*p* < .001), less psychological distress (*p* < .001), and less suicide ideation (*p* < .001).

Hypothesis two (b): PCEs will lessen the negative effects of ACEs on mental health outcomes

To test the second part of hypothesis two, PCEs score were added to model where ACEs predicted mental health outcomes. Results from these adjusted models indicated that the associations between ACEs and psychological distress and suicide ideation remained significant; however, the strength of their slope estimates and their significance levels decreased (*p* < .05 and *p* < .01 respectively). In the case of resilience, the relationship with ACEs did not remain significant (see Table 3). It thus appears that the inclusion of PCEs in the model indicates a potential compensatory effect that PCEs scores have on ACE scores.

Hypothesis three: The relationship between ACEs and mental health outcomes will be weaker among participants with higher PCEs

Lastly, to investigate whether PCEs moderate the effects of ACEs on college students’ mental health functioning, the sample was stratified by PCEs into two groups using mean split (≤8.0 vs. >8.0) and regression analyses were conducted. The relationships between ACEs and poorer mental health outcomes were stronger among those with less than or equal to the average PCEs (e.g., ≤8) as compared to those with more than the average number of PCEs (e.g., >8) (see Table 4). Thus, in support of the protective model of resilience, having higher than average cumulative PCEs are advantageous for those experiencing ACEs, in terms of effects on mental health outcomes.

**Table 4.** Protective Factor Model: Regression Results for Mental Health Outcomes in the Sample of College Students (*N* = 321).

	Stratified sampling by PCEs	
	≤8 *n* = 171	>8 *n* = 150
Outcome variables	ACEs	ACEs
Psychological distress	0.23^**^	0.15^^^
Suicide ideation	0.25^***^	0.17^*^
Resilience	−0.21^**^	−0.09^ns^

*Note*. Independent variable = PCEs and control for gender; ns = not significant; ACE = adverse childhood experience; PCE = positive childhood experience.

^ *p* < .10. *p < .05. **p < .01. ***p ≤ .001.

## Discussion

The present study contributes to a growing body of research showing that ACEs are common in college students and associated with poorer mental health outcomes, namely psychological distress, suicide ideation, and reduced resilience. It thus appears that a significant number of young people enter college with prior potentially traumatic experiences that can impact upon their academic and social lives (Karatekin, 2018; Qu et al., 2022). However, this study also contributes to developing research into the long-term correlates of PCEs and highlights that PCEs are also common among college students, with 90% reporting at least four of the ten positive experiences from their childhood. A similarly high proportion of young adults from a general population sample in the United States have reported multiple PCEs (Bethell et al., 2019). In the present study, PCEs were found to be negatively associated with psychological distress and suicide ideation, and positively associated with resiliency.

Further to this, and in support of the compensatory model of resilience, PCEs were found to have an independent, direct, and positive association with students’ mental health functioning. They also somewhat offset the negative effects of ACEs on students’ mental health, so that when PCEs were considered in a regression model, the relationships between ACEs and poorer mental health outcomes weakened. In support of the protective model of resilience, the present study found that there was a weaker relationship between ACEs and negative outcomes among individuals with higher than average reported PCEs, when compared with those whose PCEs count was the group average or less. Consequently, it is proposed that PCEs can have both compensatory and protective roles to play in the long-term effects of ACEs on mental health.

Both risk and protective factors shape children’s present and future lives, influencing their health, social connections, and relationships (Bethell et al., 2019; Crouch et al., 2019). Findings from the present study highlight the importance of PCEs on later well-being and place value on creating and implementing strategies and policy frameworks that recognize the importance of these early positive experiences (Sege & Harper Browne, 2017). Eliminating certain ACEs, such as severe illness or death of a family member, is not possible. Instead, maximizing PCEs may be a more practical and achievable goal. PCEs can mitigate the harmful effects of ACEs by supporting children in building resilience when faced with difficulties.

Given this affirmative influence of PCEs on mental health in college students, the deficiency of such advantageous experiences may be harmful in and of itself. For students who report few PCEs, a supportive environment in college can be helpful. As such, university counseling services, well-being officers, and college committees should work together with students’ unions and other student-led bodies to create a positive, well-being-focused culture in lectures, labs, and social gatherings.

From a practice perspective, practitioners and college counselors working with college students should be aware of the potential impact of ACEs on mental health and consider incorporating screening for childhood trauma into their assessments. Additionally, they may want to focus on building resilience and coping skills in college students who have experienced childhood trauma, as well as promoting positive experiences to mitigate the impact of ACEs on mental health.

ACEs do not determine the fate of an individual’s present or future life. Our brains respond differently depending on the presence and balance of both risks and protective factors that we are exposed to during our formative years (Luthar & Cicchetti, 2000; Rutter, 1985). Resilience holds a key to helping us when we are in a predicament and PCEs provides support in helping us recognize and understand our strengths and vulnerabilities. Future research aimed at understanding the impact of protective factors in our lives could examine mental health functioning by specific ACE exposures such as abuse, neglect, or household dysfunction. It would also be worthwhile to stratify participants by some demographic factors such as gender, sexual orientation, socioeconomic status, and cultural differences in order to learn more about how these protective factors operate. Additionally, future studies should also consider the timing and duration of adverse and positive childhood events to better understand the interaction of positive and adverse experiences on later mental health. For example, ACEs experienced during adolescence can have a greater effect on health than ACEs experienced in early or middle childhood (Flaherty et al., 2013).

### Limitations

Despite the significant contributions of the present study and a detailed evaluation of the independent and interactive relationship of ACEs and PCEs on college students’ mental health, some limitations should also be noted. First, the study has restricted generalizability as the sample was largely western and female. Future research could replicate and confirm the model from this study in a wider, more diverse population. Another challenge in generalizing these findings is that convenience sampling was used in the present study, as all individuals who volunteered to participate in the research were allowed to do so.

The limited nature of data on PCEs is also worth noting. Future studies should aim to integrate a broader umbrella of these types of experiences such as supportive environments, safe space within the social environment, participation in activities (volunteering, sports, etc.), or personal achievements and accomplishments. In a similar vein, future studies should also look at a broader classification and conceptualization of ACEs to include pertinent issues for particular populations such as cyberbullying or community violence.

In terms of analysis, future research could also test for an interaction between the total ACE score and the total PCE scores in order to examine if promotive factors and resources moderate the relationship between identified risk factors and outcome variables. Implementing this refined approach to testing the data could extend our empirical understanding.

The potential for ACE or PCE items to overlap with items from the other scales used in this study should also be considered and addressed in future studies. For example, even though they ask about different time frames, both the PCE scale and the CORE-10 ask participants about their experiences of social support. This can unintentionally contribute to the strength of association found between the two measures and is best avoided.

## Conclusions

As society continues to attempt to address the causes of childhood adversities, attention must also be given to the creation of those positive early experiences that both reflect and generate resilience. This study contributes to the existing literature that highlights the prevalence of ACEs, PCEs, and their associations with mental health outcomes among college students. Although ACE exposure increases the risk of mental health issues, PCEs can moderate this association, while also being associated with better mental health outcomes in their own right. Understanding the long-term correlates of ACEs and PCEs among college students and full engagement with higher education sectors can help in improving assessments of student needs and providing targeted interventions. Engaging with students to acknowledge the adversities they face but also leveraging existing strengths is one important way forward.
